# Sulcal depth-based individualized structural covariance networks reveal multi-level brain reorganization associated with cognitive performance in COPD

**DOI:** 10.3389/fnins.2026.1911246

**Published:** 2026-08-21

**Authors:** Jiajie Chen, Yanrong Chen, Kun Zhang, Kai Xu, He Hei, Wenli Huo, Wei Sheng, Pardis Bahadori, Guangming Ma, Chenwang Jin

**Affiliations:** 1 Department of Radiology, The First Affiliated Hospital of Xi’an Jiaotong University, Xi’an, China; 2 Department of Radiology, Baoji Central Hospital, Baoji, China; 3 MR Research Collaboration, Siemens Healthineers, Shanghai, China; 4 Department of Radiology, Affiliated Hospital of the Shaanxi University of Chinese Medicine, Xianyang, China

**Keywords:** chronic obstructive pulmonary disease, cognitive impairment, graph theory, individualized structural covariance network, sulcal depth

## Abstract

**Background:**

Chronic obstructive pulmonary disease (COPD) is frequently complicated by cognitive impairment, yet the underlying large-scale structural network alterations remain poorly understood. This study aimed to investigate individualized structural covariance networks (ISCNs) based on sulcal depth in COPD patients and to explore their relationship with cognitive performance.

**Methods:**

Seventy-two patients with stable COPD and 68 age- and sex-matched healthy controls underwent 3T T1-weighted MRI. ISCNs were constructed using Jensen–Shannon divergence-based similarity of sulcal depth across 148 cortical regions. Graph-theoretical metrics, network-based statistics, and partial least squares regression were employed to characterize network topology, identify altered connectivity, and examine network associations with cognition assessed via Montreal Cognitive Assessment (MoCA) scores.

**Results:**

COPD patients exhibited region-specific, bidirectional sulcal depth changes. At the network level, morphological similarity was reduced between the cingulo-opercular network and both the ventral multimodal and frontoparietal networks, whereas default mode–auditory network similarity was increased. Graph analysis revealed significantly higher gamma and lambda in COPD patients, indicating excessive local modularity alongside impaired global integration. Nodal efficiency was widely reduced in bilateral insular and frontal regions, but paradoxically increased in the left superior circular sulcus of the insula and right anterior cingulate gyrus. Partial least squares regression identified 14 interregional connections that collectively explained 28.3% of the variance in MoCA scores, with both positively and negatively weighted connections.

**Conclusion:**

COPD is associated with a multi-level reorganization of brain structural networks, spanning from local sulcal depth abnormalities to global topological imbalance and bidirectional connectivity changes. These bidirectional network shifts may be associated with the cognitive impairment in this population, offering novel network-based biomarkers for early detection and intervention.

## 1 Introduction

Chronic obstructive pulmonary disease (COPD) is a common chronic respiratory disorder characterized by persistent respiratory symptoms and progressive airflow limitation. Globally, an estimated 213.4 million prevalent cases were reported in 2021 (Wang Z. et al., 2025), and the disease remains the third leading cause of death worldwide (de Oca et al., 2025). Beyond its pulmonary manifestations, COPD is increasingly recognized for its impact on cognition functions. Studies report that approximately 32% of COPD patients exhibit some degree of cognitive impairment (Pierobon et al., 2018), a figure that rises to 56% with comprehensive neuropsychological testing (Cleutjens et al., 2018). These deficits typically involve executive function, attention, and memory, and are linked to reduced daily functioning, poorer overall health, and a higher risk of hospitalization (von Siemens et al., 2019). Although COPD-related pathophysiological factors are thought to contribute to structural brain damage over time, the extent to which these changes manifest as coordinated structural brain network alterations remains poorly understood the exact mechanisms remain unclear.

Neuroimaging studies reveal widespread gray matter abnormalities in COPD, including within the frontal, temporal, occipital, and insular regions (Chen et al., 2026; Fukatsu-Chikumoto et al., 2024; Hu et al., 2018; Wang et al., 2017), with meta-analytic evidence further corroborating consistent alterations in the postcentral gyrus, precentral gyrus, and cingulate gyrus (Liang et al., 2025; Wang et al., 2023). However, these region-level analyses treat brain regions as independent units and cannot capture how morphological changes are coordinated across distributed networks, leaving the system-level organization of gray matter largely unexplored. Individualized structural covariance networks (ISCNs) address this limitation by capturing coordinated patterns of cortical morphology at the individual level, enabling assessment of brain organization beyond single regions. Although ISCNs have been employed in other neurological and psychiatric conditions (Fan et al., 2025), their application in COPD remains entirely unexplored.

Sulcal regions are supplied by deep perforating arteries with limited collateral blood supply, making them vulnerable to the chronic hypoxia and microvascular changes that characterize COPD. This biological specificity positions sulcal depth as a mechanistically motivated morphological index for investigating COPD-related brain changes (Häkkinen et al., 2025). Sulcal depth reflects the geometry of cortical folding, which is shaped by both tissue integrity and the underlying white matter and vascular architecture, and is therefore more directly informative about the structural consequences of hypoxic–ischemic injury than cortical thickness or gray matter volume (Essen, 1997). Sulcal depth also exhibits high test–retest reliability and relative resilience to scanner and sequence variability (Pizzagalli et al., 2020). Furthermore, sulcal architecture provides the structural scaffold upon which large-scale functional networks are organized (Xiao et al., 2024), and sulcal depth variations have been associated with cognitive performance (Lin et al., 2021). Despite this, no study has exployed sulcal depth as the morphological basis for constructing individualized structural covariance networks in COPD, and whether this approach can reveal network-level reorganization inaccessible to conventional regional analyses remains unknown.

To address these gaps, the present study investigated individualized structural covariance networks derived from sulcal depth in patients with COPD. We characterized both global and regional network topologies and evaluated their associations with cognitive performance, hypothesizing that COPD patients would exhibit distinctive network topology and altered interregional morphological similarity, correlating with cognitive impairment.

## 2 Materials and methods

### 2.1 Participants

Patients with stable COPD and healthy controls (HCs) were recruited from the Affiliated Hospital of Shaanxi University of Chinese Medicine and Baoji Central Hospital. COPD diagnosis was confirmed according to the 2025 GOLD guidelines. Inclusion criteria were Han Chinese ethnicity, right-handedness, an age range of 50–80 years, and at least 6 years of education. Exclusion criteria included psychiatric disorders as diagnosed by the Diagnostic and Statistical Manual of Mental Disorders, Fifth Edition (DSM-5), recent participation in a clinical trial (within the past 3 months), severe systemic comorbidities, substance abuse, or inability to complete neuropsychological assessments.

HCs were recruited from both community and hospital sources and were matched to COPD patients based on sex and education. This study was approved by the Ethics Committee of the Affiliated Hospital of the Shaanxi University of Chinese Medicine [Approval Number: SZFYIEC-PJ-2021 No. (233)]. All participants provided written informed consent after receiving a detailed explanation of the research protocol.

### 2.2 Clinical and cognitive assessments

Pulmonary function in COPD patients was evaluated using standard spirometry, including measurements of forced vital capacity (FVC), forced expiratory volume in one second (FEV_1_), and the ratio of FEV_1_ to FVC (FEV_1_/FVC). Cognitive performance was assessed with the Montreal Cognitive Assessment (MoCA).

### 2.3 Imaging protocol

All participants underwent 3T MRI scanning at two centers. Site 1 (Affiliated Hospital of Shaanxi University of Chinese Medicine) used a Siemens MAGNETOM Skyra scanner with a 32-channel head coil, and Site 2 (Baoji Central Hospital) used a GE DISCOVERY MR750w scanner with an 8-channel head coil. 3D T1-weighted sequence parameters were as follows: Site 1—repetition time (TR) = 2,300 ms, echo time (TE) = 2.9 ms, inversion time (TI) = 900 ms, flip angle = 9°, field of view (FOV) = 240 × 240 mm^2^, matrix = 256 × 256, number of excitations (NEX) = 1, voxel size of 1 mm isotropic; Site 2—TR = 8.6 ms, TE = 3.1 ms, TI = 450 ms, flip angle = 12°, FOV = 240 × 240 mm^2^, matrix = 256 × 256, NEX = 1, voxel size of 1 mm isotropic. All images were independently reviewed by two board-certified neuroradiologists blinded to participant group. Images with excessive motion artifacts or structural abnormalities were excluded.

### 2.4 MRI processing and network construction

Structural 3D T1-weighted MRI data were processed using CAT12.9 and SPM12 (MATLAB 2024a). The preprocessing pipeline included tissue segmentation into gray matter (GM), white matter (WM), and cerebrospinal fluid (CSF), followed by DARTEL-based spatial normalization to MNI152 template space. Global measurements, including total intracranial volume (TIV), GM, WM, and CSF volumes, were derived. Sulcal depth was computed as the Euclidean distance between the central cortical surface and its convex hull. Individual sulcal depth maps were smoothed using a Gaussian kernel with a full width at half maximum of 12 mm. Regional sulcal depth values were then extracted based on 148 regions of interest from the Destrieux atlas.

To mitigate site-related variability in scanners and acquisition parameters, ComBat harmonization was applied using the sva package (v3.34.0) in R (v3.6.1), which employs an empirical Bayesian framework to model and remove site effects while preserving biological variance associated with age, sex, and diagnostic group.

ISCNs were constructed at both regional and network level. At the regional level, a 148 × 148 network was built for each subject using a Jensen–Shannon divergence (JSD)-based similarity measure by estimating interregional morphological similarity among all regions of interest (ROIs) defined by the Destrieux atlas (Li et al., 2023; Yi et al., 2022). For each ROI, the probability distribution of the morphological feature was estimated via kernel density estimation after regressing out the effects of total intracranial volume, age, and sex. The JSD between each pair of ROIs was then calculated and converted to a similarity value. At the network level, the 148 ROIs were categorized into 12 large-scale functional networks (Fan et al., 2025): primary visual (VIS1), secondary visual (VIS2), somatomotor network (SMN), cingulo-opercular network (CON), dorsal attention network (DAN), language network (LAN), frontoparietal network (FPN), auditory network (AUD), default mode network (DMN), posterior multimodal network (PMM), ventral multimodal network (VMM), and orbital affective network (ORA). A corresponding 12 × 12 ISCN was subsequently constructed for each subject by averaging JSD-based regional similarities of connections within and between each of these functional networks.

Binary networks were generated over a range of sparsity thresholds from 0.05 to 0.50 with an interval of 0.05. This sparsity range was selected to ensure that all networks remained fully connected (no isolated nodes) while preserving small-world organization, confirming that the network topology did not degrade into a random or fragmented configuration across the entire thresholding spectrum. Graph-theoretical metrics, including global small-world parameters (sigma, lambda, gamma) and nodal efficiency, were computed using GRETNA software. Area under the curve (AUC) was calculated for each metric across all sparsity levels for subsequent statistical analyses.

### 2.5 Statistical analysis

Demographics and clinical characteristics were compared between groups using independent samples *t*-tests for normally distributed continuous variables (verified by Shapiro-Wilk test, *P* > 0.05), Mann-Whitney U tests for non-normally distributed continuous variables, and chi-square tests for categorical variables. Continuous data are presented as mean ± standard deviation or median (interquartile range) as appropriate, and categorical data as frequencies and percentages. Statistical significance was set at *P* < 0.05. Neuroimaging group differences were evaluated with nonparametric permutation tests (10,000 permutations), controlling for age, sex, smoking and education. False discovery rate (FDR, *q* < 0.05) was applied separately to different metric categories. Network-based statistics (NBS) identified interregional similarity differences using a primary edgewise threshold of *P* < 0.0001. Component significance was assessed via permutation-derived null distributions. Inter-subnetwork analyses used 12 × 12 mean similarity matrices. Graph metrics and regional morphology measures were tested similarly, with FDR correction applied to global vs. nodal metrics and to each morphological index separately.

### 2.6 Morphology-cognitive associations

To examine the multivariate relationship between sulcal depth-based morphological features and cognitive performance, we employed partial least squares regression (PLSR) with MoCA scores as the dependent variable. Feature selection was restricted to those interregional connections and nodal efficiency measures that exhibited significant between-group differences in the preceding graph-theoretical and network-based analyses. Age, years of education, and total intracranial volume (TIV) were entered as covariates and regressed out before model fitting.

Model complexity was determined using leave-one-out cross-validation (LOOCV) with a maximum of 10 components. Collinearity among predictors was assessed; features with a Variance Inflation Factor (VIF) > 5 were excluded to ensure model stability. For each potential number of components, we computed the root mean squared error of prediction (RMSEP) and predicted residual sum of squares (PRESS). The optimal number of components was selected based on the minimum RMSEP. The cross-validated proportion of variance explained by the final model (Q^2^) was derived from the PRESS statistic.

To further characterize individual feature contributions, we calculated Pearson correlations and fitted simple linear regressions between each morphological feature and MoCA scores, with the coefficient of determination (R^2^) used to quantify explained variance at the single-feature level.

## 3 Results

### 3.1 Baseline demographic characteristics

A total of 140 participants were included, comprising 72 patients with COPD and 68 healthy controls (HCs) from two sites. COPD patients were significantly older than HCs (*P* < 0.001). No significant differences were observed in years of education, sex distribution, smoking history, total intracranial volume, gray matter volume, or white matter volume (all *P* > 0.05; Table 1). Gray matter volume was lower in COPD patients than HCs (*P* = 0.04), and CSF volume was significantly higher in the COPD group (*P* = 0.005). COPD patients demonstrated significantly lower MoCA scores compared to HCs (*P* < 0.001) (Table 1).

**TABLE 1:** Demographic and physiological characteristics between COPD and HC group.

Characteristics	COPD (*n* = 72)	HC (*n* = 68)	*t/x*^2^ */U*	*P*
Age (years)	62.6 ± 8.8	54.9 ± 8.2	5.36	< 0.001
Education (years)	10.5 ± 1.7	11.3 ± 3.0	–1.65	0.1
Gender (Male/Female)	61/11	56/12	0.02	0.88
Smoking (pack-years)	39.3 (24.9, 50.1)	27.0 (23.5, 43.5)	1305	0.45
TIV (cm^3^)	1500.4 ± 131.1	1472.2 ± 162.6	1.13	0.26
Gray matter (cm^3^)	626.7 ± 46.0	645.9 ± 59.2	–2.14	0.04
White matter (cm^3^)	502.9 ± 59.5	495.0 ± 53.8	0.83	0.41
CSF (cm^3^)	370.9 ± 77.7	331.2 ± 86.6	2.86	0.005
MoCA	21.7 ± 3.7	26.8 ± 0.8	–11.11	< 0.001

COPD, Chronic Obstructive Pulmonary Disease; HC, Healthy Control; TIV, total intracranial volume; MoCA, Montreal Cognitive Assessment; CSF, Cerebrospinal Fluid. Continuous variables are presented as mean ± standard deviation or median (interquartile range) for non-normally distributed variables.

### 3.2 Validation of ComBat harmonization

To verify the efficacy of ComBat harmonization, we compared sulcal depth distributions across sites and diagnostic groups before and after adjustment. Before harmonization, the median sulcal depth was 0.32 [interquartile range (IQR) = 0.28–0.35] for the pooled sample. After ComBat adjustment, the median decreased to 0.29 (IQR = 0.27–0.32), with a narrower interquartile range suggesting reduced inter-site variability. Between-site differences were not significant either before or after harmonization (all *P* > 0.05), and within-site group comparisons (COPD vs. HC) remained unchanged (*P* = 0.29 and 0.47 for the two sites), confirming that the harmonization removed potential scanner-related variance while preserving the biological signal of interest. Detailed validation statistics are provided in Supplementary Table 1.

### 3.3 Group-level alterations in sulcal depth

**FIGURE 1:**
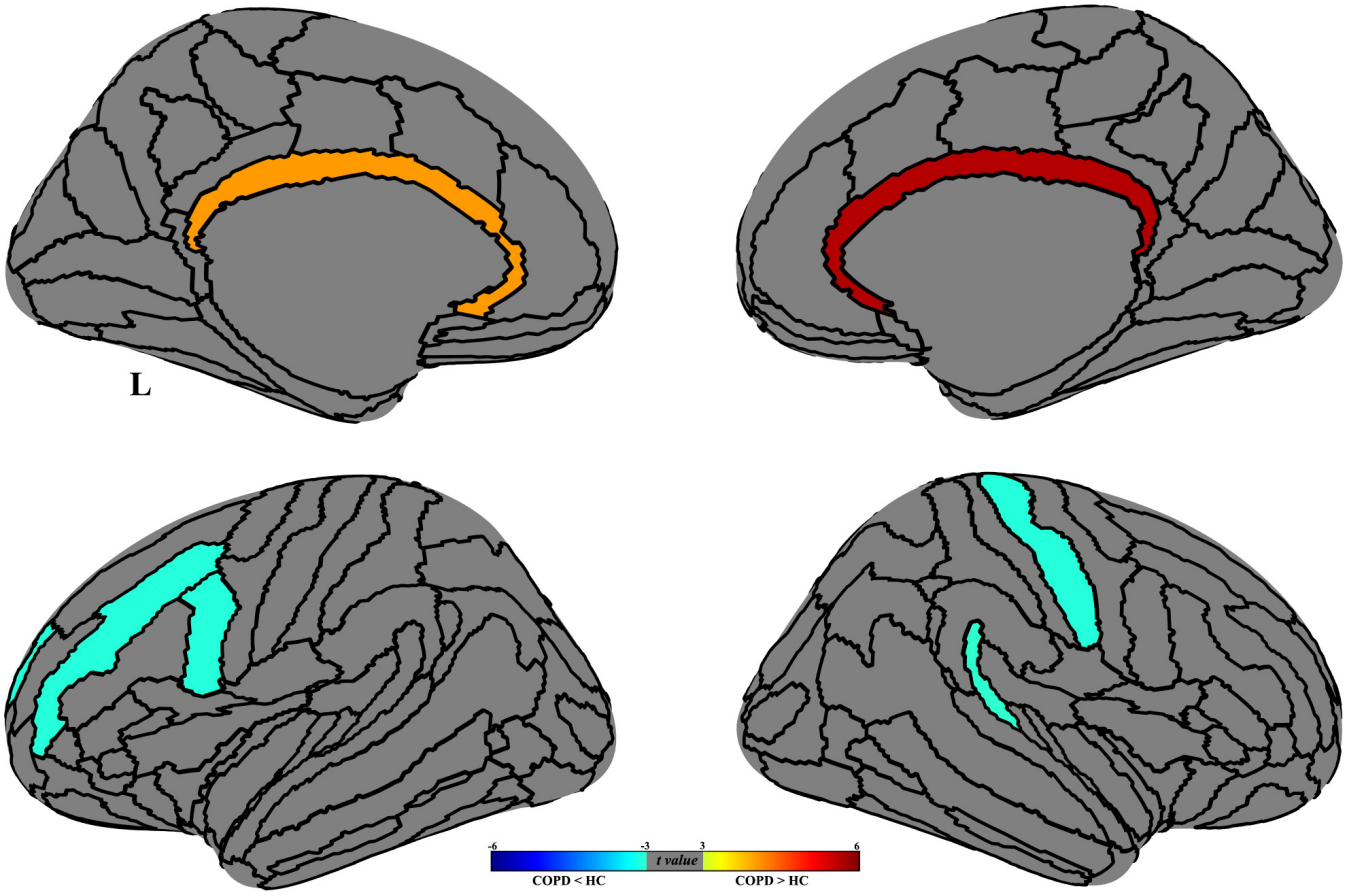
Significant differences in sulcal depth between COPD and HCs.

Group comparison revealed significant differences in sulcal depth between COPD patients and HCs (Figure 1). Patients with COPD showed greater sulcal depth in the bilateral superior pericallosal sulcus compared to HCs. Conversely, reduced sulcal depth was observed in the left middle frontal gyrus, left inferior part of the precentral sulcus, and right central sulcus (FDR-corrected *p* < 0.05).

### 3.4 Inter-subnetwork and interregional morphological similarity

**FIGURE 2:**
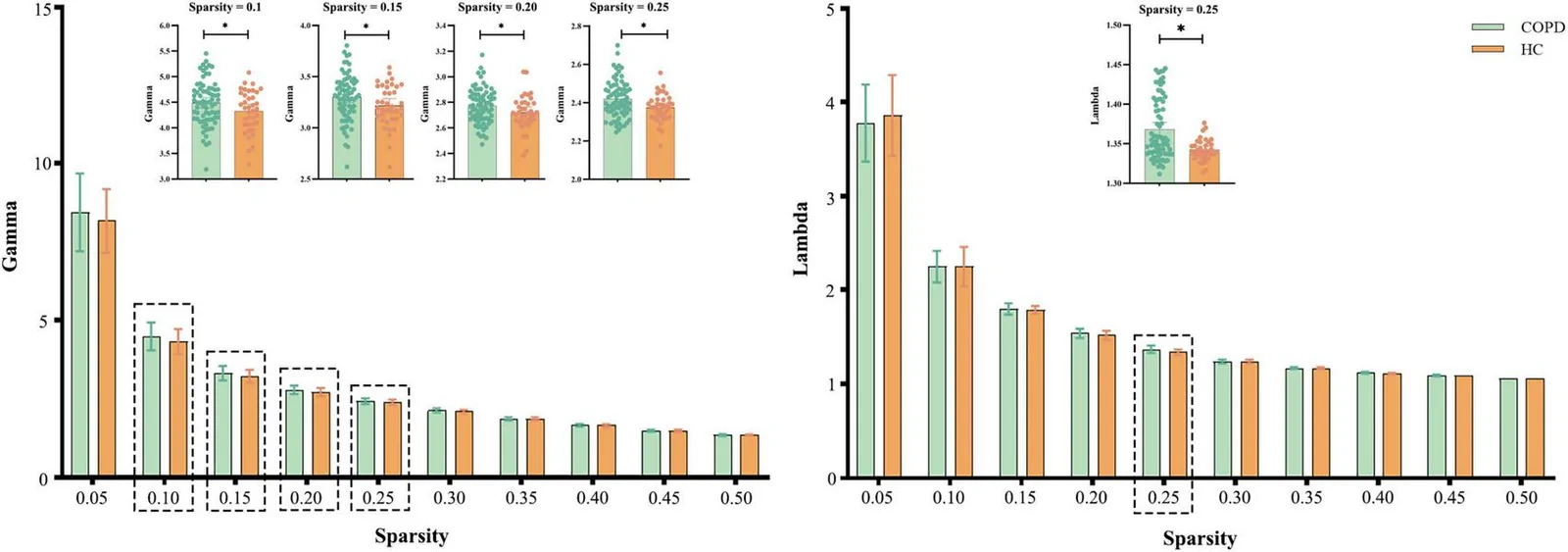
Between-group differences in inter-subnetwork morphological similarity. *Indicates statistical significance at *p* < 0.05 (two-tailed).

Compared with the HCs, patients with COPD exhibited significantly altered morphological similarity at both the inter-subnetwork (Figure 2) and interregional levels. Decreased similarity was observed between the cingulo-opercular network and both the ventral multimodal and frontoparietal networks, as well as between the default mode network and the cingulo-opercular network. Conversely, increased morphological similarity was observed between the default mode network and the auditory network.

**FIGURE 3:**
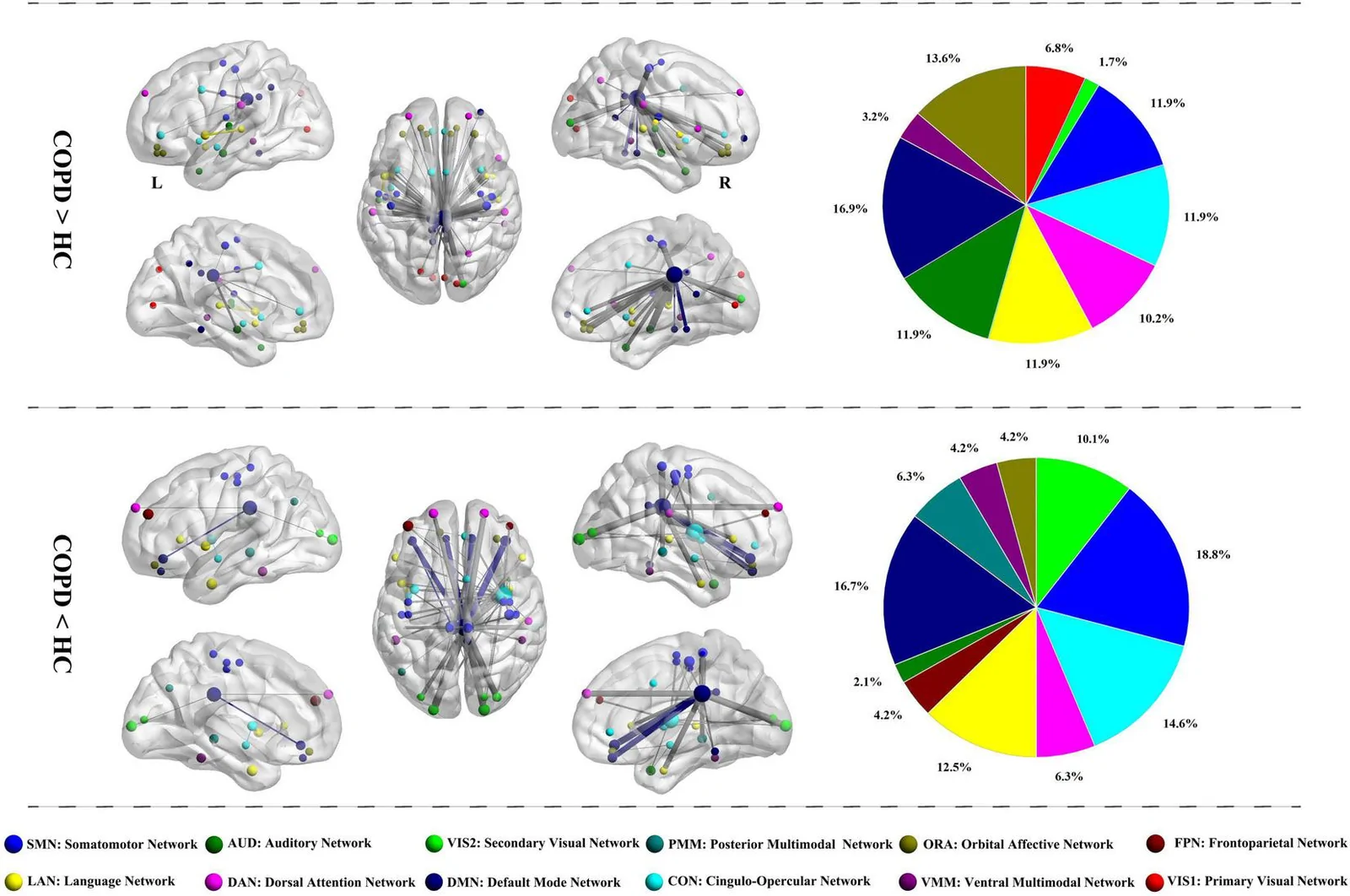
Alterations in global gray matter network topology between COPD and HC groups.

At the interregional level, 130 connections with significant changes between the two groups were identified (Figure 3). The COPD group exhibited increased morphological similarity in a connected subnetwork comprising 59 nodes and 70 connections. These connections were distributed primarily across the default mode (16.9%), orbital-affective (13.6%), cingulo-opercular (11.9%), language (11.9%), auditory (11.9%), somatomotor (11.9%), dorsal attention (10.2%), primary visual (6.8%), secondary visual (1.7%), and ventral multimodal (3.2%) networks. Conversely, the COPD group showed decreased morphological similarity in a second component consisting of 48 nodes and 60 connections. These were mainly distributed in the somatomotor (18.8%), default mode (16.7%), cingulo-opercular (14.6%), language (12.5%), secondary visual (10.1%), dorsal attention (6.3%), posterior multimodal (6.3%), frontoparietal (4.2%), ventral multimodal (4.2%), orbital-affective (4.2%), and auditory (2.1%) networks.

### 3.5 Network topology and nodal efficiency

**FIGURE 4:**
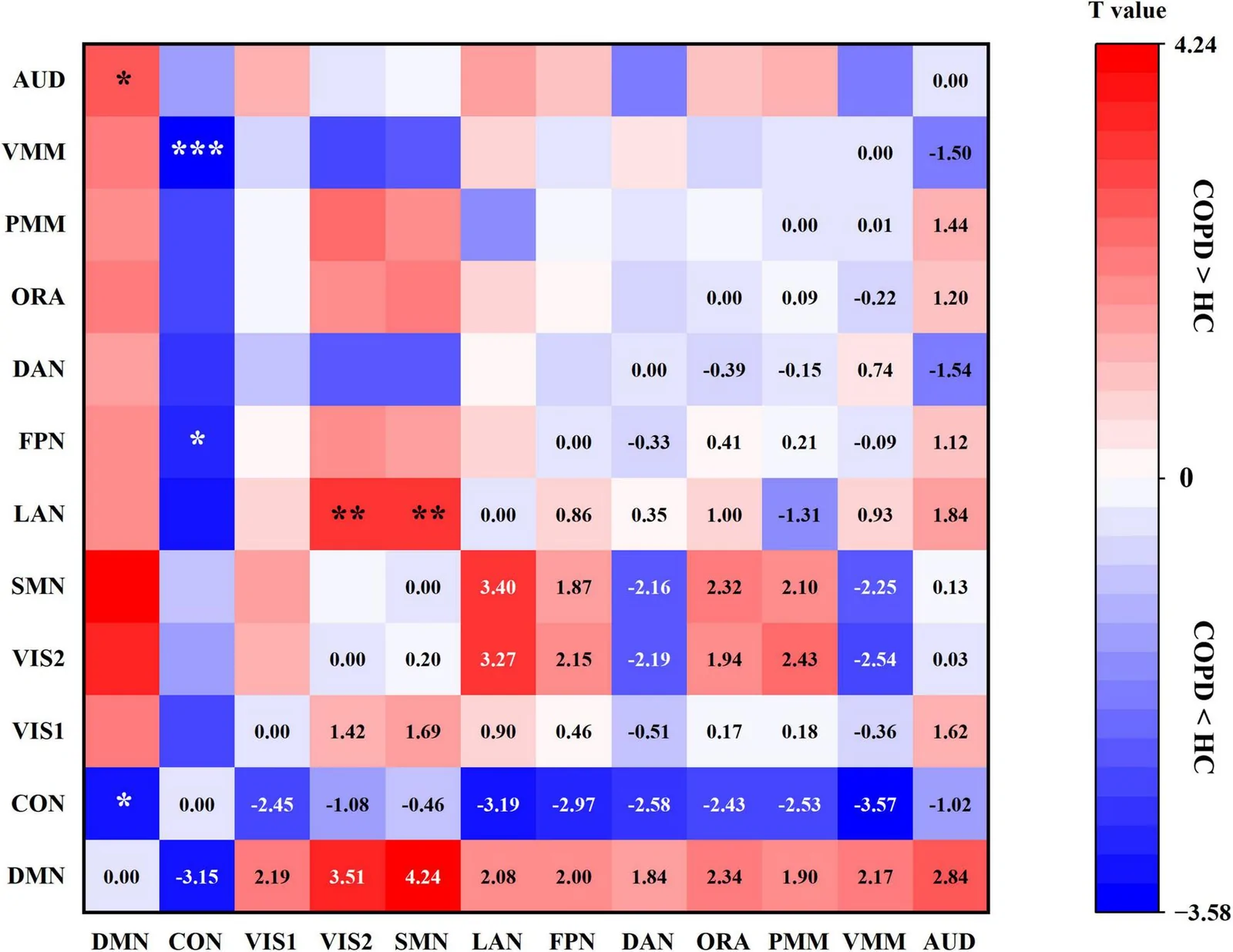
Comparison of small-world properties in gray matter morphological networks between COPD and HCs. Statistical significance levels: *p < 0.05; **p < 0.01; ***p < 0.001.

Both groups exhibited small-world properties in their individual structural covariance networks (γ > 1, λ≈ 1, σ > 1). However, compared to the HC group, the COPD group demonstrated a significantly higher normalized clustering coefficient (γ) at network densities ranging from 0.10 to 0.25 (*P* < 0.05, FDR corrected). A significantly higher normalized characteristic path length (λ) was also observed in the COPD group at a network density of 0.25 (*P* < 0.05, FDR corrected). No significant between-group differences were found for small-worldness (σ) across the tested densities tested (Figure 4).

**FIGURE 5:**
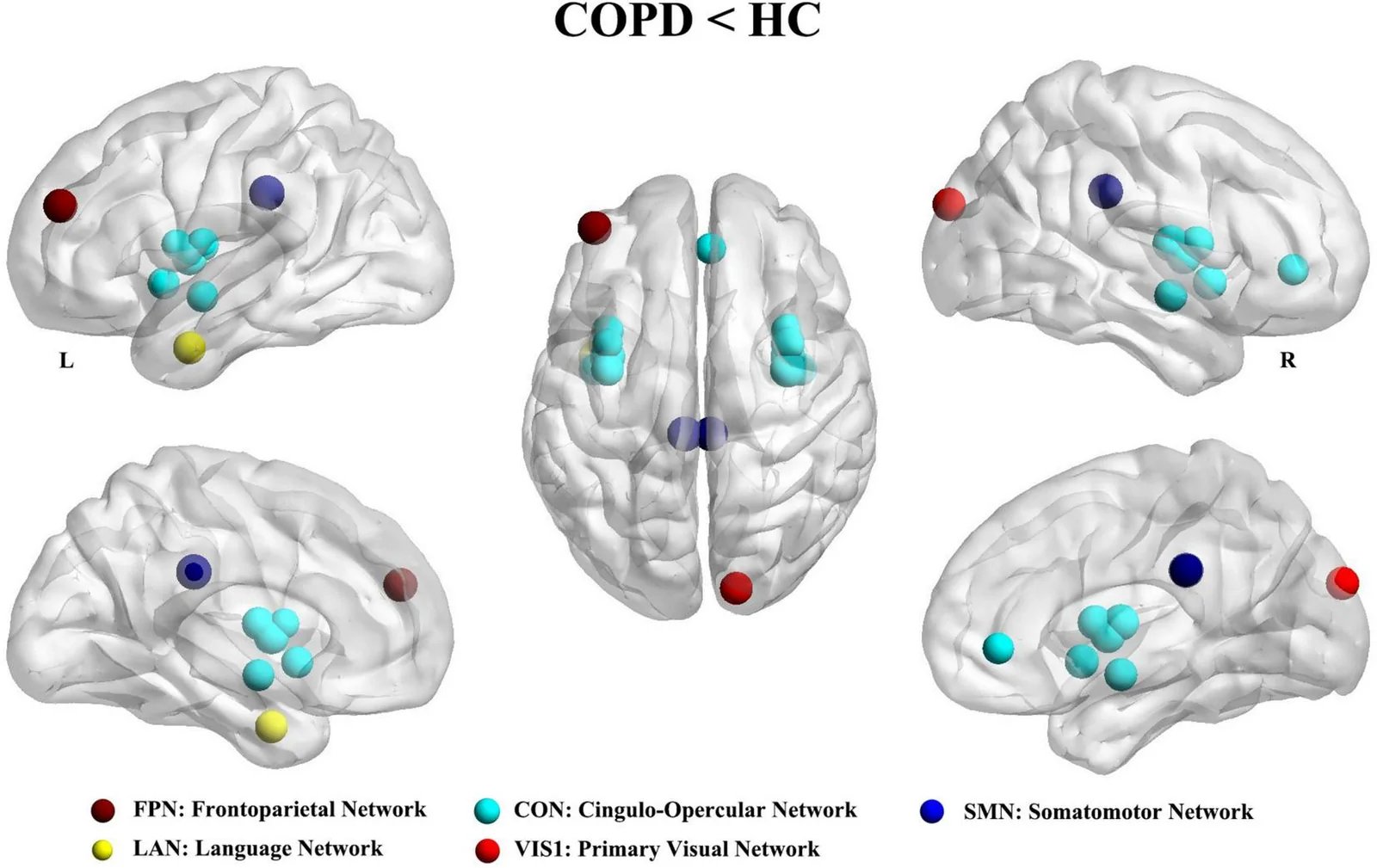
Between-group differences in nodal efficiency.

Graph-theoretical analysis revealed significant between-group differences in nodal efficiency across 16 cortical regions (Table 2 and Figure 5). Fourteen regions showed significantly reduced nodal efficiency in COPD patients compared with HCs, distributed across bilateral insular, frontal, temporal, and occipital regions, including the circular sulci of the insula, insular gyri, middle frontal gyrus, pericallosal sulcus, and temporal pole (all *p* < 0.05, FDR corrected). Against this widespread reduction, two regions exhibited significantly increased nodal efficiency in COPD patients: the left superior circular sulcus of the insula (*t* = 3.92, *P* = 0.008, FDR corrected) and the right anterior cingulate gyrus and sulcus (*t* = 3.91, *p* = 0.009, FDR corrected).

**TABLE 2:** Between-group differences in nodal efficiency (COPD vs. HC).

Region	COPD	HC	*t*	*P*
Left anterior circular sulcus of the insula	0.52 ± 0.02	0.54 ± 0.02	–3.01	0.031
Left inferior circular sulcus of the insula	0.13 ± 0.05	0.16 ± 0.02	–2.86	0.046
Left long insular gyri and central insular sulcus	0.16 ± 0.06	0.2 ± 0.04	–3.32	0.015
Left middle frontal gyrus	0.14 ± 0.05	0.18 ± 0.03	–3.56	0.009
Left pericallosal sulcus	0.14 ± 0.05	0.18 ± 0.03	–3.80	0.008
Left short insular gyri	0.14 ± 0.06	0.18 ± 0.03	–3.75	0.009
Left temporal pole	0.14 ± 0.05	0.18 ± 0.03	–3.19	0.021
Right anterior circular sulcus of the insula	0.53 ± 0.02	0.54 ± 0.02	–3.38	0.014
Right inferior circular sulcus of the insula	0.15 ± 0.06	0.19 ± 0.03	–3.14	0.022
Right long insular gyri and central insular sulcus	0.13 ± 0.05	0.17 ± 0.03	–4.05	0.008
Right pericallosal sulcus	0.13 ± 0.05	0.16 ± 0.03	–3.58	0.009
Right short insular gyri	0.51 ± 0.01	0.54 ± 0.01	–3.62	0.009
Right superior circular sulcus of the insula	0.13 ± 0.05	0.16 ± 0.03	–3.39	0.014
Right superior occipital gyrus	0.51 ± 0.02	0.53 ± 0.02	–3.32	0.015
Left superior circular sulcus of the insula	0.54 ± 0.02	0.52 ± 0.02	3.92	0.008
Right anterior cingulate gyrus and sulcus	0.54 ± 0.02	0.52 ± 0.03	3.71	0.009

### 3.6 Brain-cognitive relationships

To examine the relationship between morphological features and cognitive performance, Partial least squares regression (PLSR) was conducted with MoCA scores as the outcome variable. Based on the group comparisons, a total of 146 features (130 interregional connections and 16 nodal efficiency measures) were identified and entered as predictors after regressing out age, education, and TIV. Following collinearity screening (VIF > 5 exclusion), the optimal model determined by LOOCV comprised one latent component (LV1), which explained 28.3% of the variance in MoCA scores (*Q*^2^ = 0.283).

**FIGURE 6:**
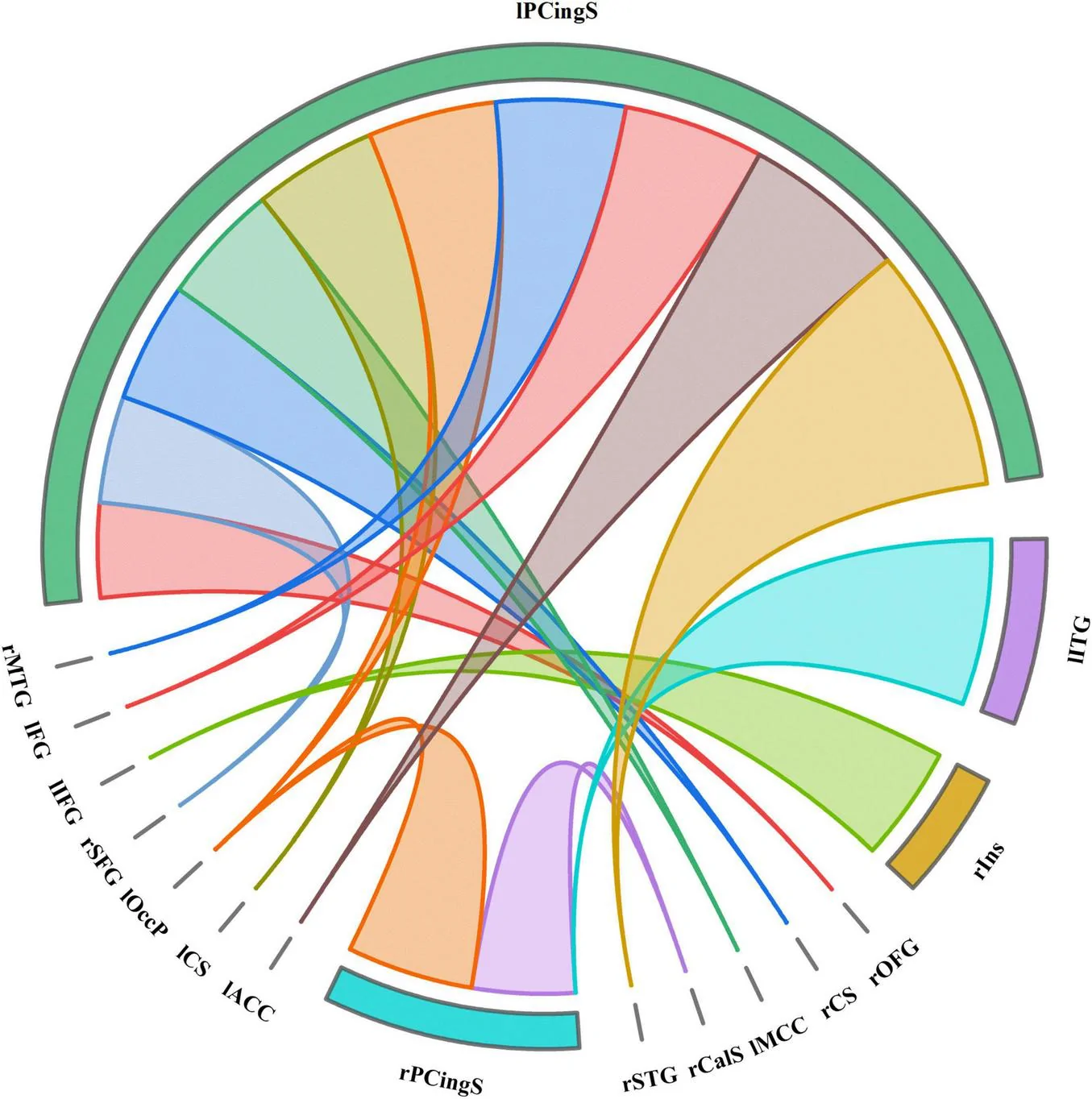
Multivariate association between brain morphological networks and cognitive performance. lACC, Left Anterior Cingulate Cortex; lCS, Left Central Sulcus; lFG, Left Fusiform Gyrus; lIFG, Left Inferior Frontal Gyrus; lITG, Left Inferior Temporal Gyrus; lMCC, Left Middle Cingulate Cortex; lOccP, Left Occipital Pole; lPCingS, Left Posterior Cingulate Sulcus; rCalS, Right Calcarine Sulcus; rCS, Right Central Sulcus; rIns, Right Insula; rMTG, Right Middle Temporal Gyrus; rOFG, Right Occipital Fusiform Gyrus; rPCingS, Right Posterior Cingulate Sulcus; rSFG, Right Superior Frontal Gyrus; rSTG, Right Superior Temporal Gyrus.

Following FDR correction (*p* < 0.05), 14 interregional connections were significantly associated with cognitive performance (Figure 6). Of these, 11 carried negative PLS weights and 3 carried positive PLS weights. Notably, the left posterior cingulate sulcus (lPCingS) featured as a node in 10 of the 14 significant connections, identifying it as the dominant structural hub linking network morphology to cognitive performance. To further characterize individual connection contributions, Pearson correlations were computed between each connection and MoCA scores; these bivariate associations are reported in Table 3 alongside PLS weights.

**TABLE 3:** Results of PLS analysis identifying interregional connections associated with MoCA scores (FDR corrected).

Interregional connection	PLS weight	*r*	*P*
lCS-lPCingS	–0.23	–0.31	0.001
rPCingS-lITG	–0.19	–0.25	0.007
rMTG-lPCingS	–0.17	–0.22	0.018
lOccP-lPCingS	–0.17	–0.22	0.019
lMCC-lPCingS	–0.16	–0.22	0.023
rSTG-lPCingS	–0.16	–0.22	0.021
lOccP-rPCingS	–0.16	–0.22	0.02
lIFG-rIns	–0.16	–0.21	0.026
rCS-lPCingS	–0.15	–0.2	0.036
rSFG-lPCingS	–0.15	–0.2	0.033
rOFG-lPCingS	–0.14	–0.19	0.045
lACC-lPCingS	0.19	0.25	0.007
lFG-lPCingS	0.17	0.23	0.014
rCalS-rPCingS	0.16	0.22	0.023

Connections with positive PLS weights, including lACC-lPCingS (*r* = 0.25), lFG-lPCingS (*r* = 0.23), and rCalS-rPCingS (*r* = 0.22), exhibited positive correlations with MoCA scores. In contrast, the 11 connections with negative PLS weights, including lCS-lPCingS (*r* = –0.31), rPCingS-lITG (*r* = –0.25), and rMTG-lPCingS (*r* = –0.22), were negatively correlated with MoCA scores. Detailed characteristics of all significant connections are presented in Table 3.

## 4 Discussion

The present study provides evidence of widespread disruptions in individual structural covariance networks derived from sulcal depth in patients with COPD. We revealed altered interregional morphological similarity, reconfigured subnetwork interactions, and disturbed topological organization in COPD patients compared to healthy controls. PLSR analysis further identified a multivariate association between these alterations and cognitive performance, explaining 28.3% of MoCA scores variance. These findings suggest that the cognitive impairment frequently observed in COPD may be underpinned by a complex pattern of large-scale structural network reorganization.

At the local morphological level, COPD patients exhibited region-specific bidirectional alterations in sulcal depth. Specifically, patients with COPD showed increased cortical depth in the bilateral superior pericallosal sulcus, whereas decreased depth was observed in the left middle frontal gyrus, left inferior part of the precentral sulcus, and right central sulcus. The middle frontal gyrus is a core node of the frontoparietal network and is involved in executive function and working memory; the precentral sulcus and central sulcus correspond to primary sensorimotor cortex. The reduced depth in these regions is consistent with previous findings of decreased gray matter volume in frontal and motor areas (Wang H. Q. et al., 2025) and reduced functional connectivity (Han et al., 2025) in COPD patients, suggesting that these changes may represent the structural correlate for impairments in executive function and motor ability. In contrast, the increased depth in the superior pericallosal sulcus may reflect a more complex interplay of mechanisms. This sulcus is adjacent to the splenium of the corpus callosum and is an important pathway for white matter tracts (e.g., the parieto-occipital bundle) connecting the two hemispheres, participating in visual information integration and interhemispheric cognitive coordination (Shah et al., 2021). Lee et al. found elevated diffusion tensor imaging metrics in the corpus callosum of COPD patients, potentially compensatory in white matter microstructural integrity (Lee et al., 2019). Nevertheless, alternative mechanical explanations deserve consideration. The increased sulcal depth may partly reflect an ex vacuo effect, in which atrophy of the adjacent cortex or underlying white matter passively enlarges the sulcal space without implying active remodeling (Bigler and Maxwell, 2011). This is particularly relevant for the superior pericallosal sulcus, which lies near the splenium and medial parietal cortices, areas vulnerable to COPD-related damage. The tension-based morphogenesis framework posits that cortical folding geometry is partially sustained by axonal tension within white matter (Essen, 1997; Ronan and Fletcher, 2015). Should COPD-related white matter changes perturb this tension, the consequent mechanical redistribution could passively alter sulcal depth. Critically, these mechanical and neuroplastic accounts are not mutually exclusive. The observed region-specific bidirectional pattern likely reflects a confluence of focal atrophy, altered mechanical tension, and disease-modulated vascular/neuroplastic factors. Resolving these mechanisms ultimately requires longitudinal imaging with multimodal tissue characterization. Thus, COPD-related cortical changes are not uniformly atrophic but show a region-specific pattern of coexisting increases and decreases, may encompass both active tissue remodeling and passive mechanical consequences of focal atrophy, and provide a local anatomical basis for subsequent complex network-level alterations.

Local morphological changes further extended to the network level. The present study found that patients with COPD showed significantly reduced morphological similarity between the cingulo-opercular network and the ventral multimodal and frontoparietal networks, as well as between the cingulo-opercular network and the default mode network, whereas similarity between the default mode network and the auditory network was increased. The cingulo-opercular network (including the anterior cingulate cortex and insula) is central to the interoceptive network and is implicated in the perception of respiratory discomfort (dyspnea) and emotional regulation (Hudson et al., 2025). The default mode network (DMN) is active during rest and participates in self-referential processing and memory retrieval. The frontoparietal network subserves cognitive control. The reduced connectivity among these networks may constitute a neural basis for the co-occurrence of dyspnea perception, abnormal self-referential processing, and executive dysfunction. The enhanced connectivity between the auditory network and the DMN may represent cross-modal potentially compensatory reorganization. Using Mendelian randomization, Guo et al. identified genetic associations between COPD and the DMN, frontoparietal network, and attention network (Guo et al., 2025). Han et al. reported abnormal resting-state functional connectivity patterns in the cingulate cortex, parieto-occipital lobe, superior temporal sulcus, and precentral gyrus in COPD patients, and these changes were significantly correlated with cognitive scores (MMSE) and FEV_1_(Han et al., 2025). Extending these findings from the perspective of structural similarity, our study further supports the presence of system-level connectivity disturbances across multiple large-scale brain networks in COPD.

More importantly, graph theoretical analysis revealed the potential impact of this network reorganization on global information processing capacity. We observed that the structural covariance networks of COPD patients exhibited significantly higher gamma and lambda values. These changes in graph metrics indicate an imbalance between excessive local modularity and insufficient global integration in the brain structural networks of COPD patients (Lu et al., 2017). A higher gamma reflects stronger local clustering or modularity, which may indicate tighter intra-modular connections (Bassett and Bullmore, 2017). A higher lambda may suggest that the global information transfer efficiency of the brain network is lower than that of a random network, with long-distance inter-modular communication becoming more circuitous and less efficient (Kawagoe et al., 2017). The simultaneous elevation of gamma and lambda may imply that the brain structural network in COPD is a suboptimal topology characterized by locally rigid and globally disconnected organization. Such a topological pattern may impair cognitive function by hindering the rapid and efficient cross-regional neural integration upon which cognitive processes rely. Previous EEG studies found lower global efficiency, local efficiency, and clustering coefficient in the alpha band in COPD patients (Choi and Kim, 2022), which is consistent with the trend of decreased global efficiency observed from the structural perspective in our study. This abnormal topological property may serve as a bridge linking morphological connectivity alterations to cognitive impairment.

We also found widespread reduction in nodal efficiency in COPD patients, primarily involving bilateral insular and frontal regions as well as the right anterior cingulate cortex. These regions have been consistently identified as key sites of COPD-related gray matter atrophy and functional connectivity abnormalities in multiple VBM studies (Esser et al., 2016; Jia-Kai et al., 2026; Liang et al., 2025; Wang et al., 2020) and resting-state fMRI studies (Lv et al., 2020). Notably, against a background of widespread reduction in global nodal efficiency, interregional morphological similarity showed both increases and decreases. The consistent decline in efficiency of key hubs such as the prefrontal cortex, insula, and anterior cingulate cortex likely represents the primary gray matter damage. Impairment of these core integration nodes likely weakens the global integration capacity of the network (Wu et al., 2016). In response, the brain network may undergo complex reorganization: some connections may weaken or break because the hub can no longer sustain them; other connections may be potentially strengthened in a compensatorily manner, possibly bypassing damaged nodes and rerouting information to maintain basic communication (Wu et al., 2022). Enhanced potentially compensatory functional connectivity has been associated with the preservation of cognitive function (Kim et al., 2025). In cognitively intact older adults, despite age-related hippocampal structural decline, they exhibit stronger prefrontal activation and stronger prefrontal-hippocampal connectivity (Hampstead et al., 2016). Therefore, the bidirectional changes in connection strength are not contradictory; rather, they may represent a macroscopic manifestation of both degenerative and compensatory processes triggered by deeper, more consistent network hub failure, and further longitudinal studies are needed to confirm this interpretation.

PLS analysis identified multivariate associations between network morphology alterations and cognitive performance, revealing the cognitive implications of the bidirectional nature of these network changes. Higher morphological similarity in connections such as lACC-lPCingS, lFG-lPCingS, and rCalS-rPCingS was associated with higher MoCA scores in COPD patients. These connections primarily involve the core nodes of the DMN (posterior cingulate sulcus) and regions related to emotion and visual processing; their enhancement may represent potentially compensatory remodeling that the brain may employ to maintain core cognitive resources in the face of COPD-related pathological challenges. Conversely, higher similarity in connections such as lCS-lPCingS, rPCingS-lITG, and rMTG-lPCingS was associated with lower MoCA scores. Their abnormal enhancement may disrupt normal network segregation and functional specialization, and co-occur with impaired cognitive performance. It should be noted that the PLSR model explained 28.3% of the variance in MoCA scores, indicating a moderate multivariate association between structural network morphology and cognitive performance. Nonetheless, the majority of variance remains unexplained, possibly reflecting contributions from demographics, disease severity, genetics, systemic inflammation, and other unmeasured cognitive determinants in COPD.

Previous studies have demonstrated that cognitive impairment in COPD patients affects multiple domains including attention, executive function, language, visuospatial ability, and delayed recall (Beers et al., 2018; Cleutjens et al., 2014), and that COPD patients may be at increased risk of dementia or cognitive impairment (Wang et al., 2022). Resting-state functional connectivity studies have also found significantly reduced functional connectivity within the visual network and frontoparietal network in patients with stable COPD (Wang et al., 2020). The bidirectional alterations observed in our study, involving nodes and connections across multiple subnetworks, likely reflect the coexistence and competition between compensatory remodeling and pathological dysregulation within the brain network.

Our findings delineate a multi-level neuroimaging mechanism in COPD, spanning from local morphological abnormalities (sulcal depth) to inter-network connectivity disturbances, then to global topological imbalance, and finally to cognitive performance influenced by bidirectional changes in specific connections. Chronic hypoxia (hypoxemia) (Liang et al., 2025), hypercapnia (Burgraff et al., 2019), inflammatory response (Ding et al., 2018), and blood-brain barrier disruption (Fan et al., 2024) have been widely recognized as key mechanisms underlying cognitive impairment via the “lung-brain axis.” The multi-level, bidirectional structural network reorganization we observed is consistent with these proposed pathophysiological mechanisms. Crucially, because we did not directly measure blood gas parameters (PaO_2_, PaCO_2_), inflammatory biomarkers, or markers of blood–brain barrier integrity, any attribution of the observed structural network changes to these specific mechanisms remains speculative. Establishing causal links between specific COPD-related physiological disturbances and brain network alterations will require future studies integrating multimodal blood biomarkers, arterial blood gas analysis, and neuroimaging in longitudinal designs. These findings offer a potential neural basis for understanding the association between lung disease and cognitive impairment and may inform future imaging biomarker development, though longitudinal and interventional validation is needed.

Although the COPD group was significantly older than the HC group, several lines of evidence suggest that the observed network alterations are unlikely to be solely attributable to age-related confounding. Age was rigorously controlled at multiple analytical steps, from regional morphological features to group comparisons and brain–cognitive association models. Furthermore, the bidirectional pattern of morphological and network changes contrasts with the unidirectional atrophy and sulcal widening typical of normal aging. The simultaneous elevation of gamma and lambda, indicating a topological pattern of excessive local modularity coupled with impaired global integration, also deviates form aging-related gradual declines. Collectively, these lines of evidence indicate that the observed changes are disease-related rather than purely age-driven.

Several limitations should be acknowledged. First, the cross-sectional design precludes causal inferences regarding the relationship between observed network alterations and disease progression; longitudinal studies are needed. Second, despite rigorous statistical control for age at multiple analytical levels, the significant age difference between COPD and HC groups remains a limitation, warranting validation with more tightly age-matched cohorts in future studies. Third, our study focused primarily on brain structural networks and lacked direct measurements of pulmonary function dynamics, arterial blood gases, or systemic inflammatory biomarkers; therefore, we could not directly verify *in vivo* the specific pathophysiological pathways of the “lung-brain axis” but instead based our inferences on existing literature. Furthermore, the interpretation of increased sulcal depth and elevated nodal efficiency as “compensatory” remains speculative given the cross-sectional nature of this study. Definitive characterization of compensatory mechanisms will require longitudinal studies tracking the trajectory of structural changes relative to cognitive decline.

In conclusion, this study, through individual-level brain structural network analysis, provides a novel evidence of a characteristic multi-level brain reorganization pattern in COPD, including local sulcal depth abnormalities, widespread inter-network connectivity remodeling, a global topological imbalance characterized by “local rigidity and global disconnection,” and bidirectional changes in specific network connections that are associated with cognitive function. These findings suggest that COPD-related cognitive impairment may arise from complex reorganization of brain structural networks rather than focal atrophy alone. These findings advance understanding of cognitive impairment in COPD and support future investigation of network-based biomarkers for early detection.

## Data Availability

The raw data supporting the conclusions of this article will be made available by the authors, without undue reservation.
