# Supplementary material for: Sulcal depth-based individualized structural covariance networks reveal multi-level brain reorganization associated with cognitive performance in COPD
Source: Front Neurosci. 2026 Aug 21;20:1911246. doi: 10.3389/fnins.2026.1911246 (PMC13541659; doi:10.3389/fnins.2026.1911246)
Supplement: Supplementary file 1 [file Table_1.docx]

| **Supplementary Table S1. Statistical Comparison of Sulcal Depth Across Sites and Groups Before and After ComBat Harmonization.** | | | | | |  |
| --- | --- | --- | --- | --- | --- | --- |
| Comparison | Group | Correction | *t* | *P* | Cohen’s *d* | |
| Between-site (Site 1 vs. 2) |  |  |  |  |  | |
|  | COPD | Original | –0.346 | 0.478 | 0.244 | |
|  | COPD | ComBat | –0.496 | 0.503 | 0.242 | |
|  | HC | Original | 1.025 | 0.352 | 0.389 | |
|  | HC | ComBat | 0.862 | 0.395 | 0.344 | |
| Within-site (COPD vs. HC) |  |  |  |  |  | |
|  |  | Original (Site 1) | –1.111 | 0.294 | 0.351 | |
|  |  | ComBat (Site 1) | –1.111 | 0.294 | 0.351 | |
|  |  | Original (Site 2) | 0.416 | 0.471 | 0.355 | |
|  |  | ComBat (Site 2) | 0.416 | 0.471 | 0.355 | |
| Abbreviations: COPD, chronic obstructive pulmonary disease. HC, healthy control. | | | | | |  |
